# Adhärenz bei der Anti-VEGF-Therapie – Überlegungen und praktische Empfehlungen

**DOI:** 10.1007/s00347-020-01273-5

**Published:** 2020-12-03

**Authors:** Albrecht Lommatzsch, Nicole Eter, Christoph Ehlken, Ines Lanzl, Hakan Kaymak, Alexander K. Schuster, Focke Ziemssen

**Affiliations:** 1 Augenzentrum am St. Franziskus-Hospital, Münster, Deutschland; 2 Klinik für Augenheilkunde, Universitätsklinikum Münster, Münster, Deutschland; 3 Klinik für Ophthalmologie, Universitätsklinikum Schleswig-Holstein, Campus Kiel, Kiel, Deutschland; 4 Chiemsee Augen Tagesklinik, Prien, Deutschland; 5 Makula-Netzhaut-Zentrum, Düsseldorf-Oberkassel, Deutschland; 6 Augenklinik und Poliklinik, Universitätsmedizin Mainz, Mainz, Deutschland; 7 Department für Augenheilkunde Tübingen, Universitäts-Augenklinik, Elfriede-Aulhorn-Str. 7, 72076 Tübingen, Deutschland

**Keywords:** Organisation, Optimierung, Intravitreale Injektion, Barrieren, Persistenz, Organization, Optimization, Intravitreal injection, Barriers, Persistence

## Abstract

**Hintergrund:**

Zahlreiche Studien haben eine mangelnde Therapieadhärenz als wichtigen Faktor identifiziert, der einer notwendigen Anzahl von Anti-VEGF-Behandlungen und somit einem besseren funktionellen Ergebnis entgegensteht.

**Fragestellung:**

Der Beitrag diskutiert konkrete Maßnahmen, die das Risiko einer zu späten oder zu seltenen intravitrealen operativen Medikamenteneingabe (IVOM) im Sinne einer Unterbehandlung verringern.

**Material und Methode:**

Im Rahmen einer Expertenrunde wurden relevante Parameter der Therapieadhärenz und Variablen identifiziert. Sinnvolle Abläufe strukturiert und organisatorischen Bereichen zugeordnet.

**Ergebnisse:**

Die Zusammenstellung identifizierter Einflussfaktoren und sinnvoller Maßnahmen (Organisation, Transport, Kommunikation, Motivation) ermöglicht es Behandlern, die eigene Umsetzung der IVOM-Therapie in unterschiedlichen Bereichen zu optimieren. Regelmäßige Monitoring-Maßnahmen können den Umfang von Therapiepausen und -abbrüchen identifizieren. Für konkrete Kennzahlen (IVOM pro Zeitintervall, längstes Pausenintervall, Mindestabdeckung pro Zeit, Verzögerungen) wurde eine Auswirkung auf die Entwicklung der Sehfunktion nachgewiesen. Organisatorische Maßnahmen, die Schulung von Team und Zuweisern, die redundante und iterative Informationsweitergabe an Patienten haben sich in der Erfahrung von Experten bewährt. Die feste Integration dieser Prozesse in bestehende Strukturen wird durch die Arbeit mit Checklisten erleichtert.

**Schlussfolgerungen:**

Eine Optimierung der Abläufe ist oft möglich, um die Adhärenz und somit die funktionellen Ergebnisse zu verbessern. Bisher fehlen jedoch noch interventionelle Studien, wie Adhärenz und Persistenz im deutschen Behandlungssetting erhöht werden.

## Abstract (en)

**Background:**

Numerous studies have identified a lack of treatment adherence as an important factor that often conflicts with the necessary number of anti-VEGF injections and therefore a better functional result.

**Objective:**

This article discusses approaches and possible measures to reduce the risk of late and infrequent intravitreal injections leading to the major issue of undertreatment.

**Material and method:**

In the course of an expert dialogue, relevant parameters of treatment adherence and variables were identified. Meaningful processes were structured and assigned to organizational areas.

**Results:**

The compilation of meaningful measures enables practitioners to optimize their own implementation in different areas. Regular monitoring measures can identify the extent of treatment interruption and discontinuation. For specific indicators (treatments per time interval, longest pause interval, minimum coverage per unit time, delay) an effect on the development of visual function was demonstrated. Organizational measures, training of teams and referring physicians, redundant and iterative information transfer to patients have been proven in the experience of experts. The firm integration of these processes into the structures is facilitated by working with checklists.

**Conclusion:**

An optimization of the processes is possible to improve the adherence and the functional results; however, interventional studies showing how adherence and persistence can be increased in the German treatment setting are still lacking.

Die intravitreale operative Medikamentenapplikation (IVOM) ist der derzeitige Therapiestandard bei verschiedenen neovaskulären und vaskulären Erkrankungen der Netzhaut. Für viele Patienten stellt die hohe Anzahl der Arzttermine und Behandlungen eine Herausforderung dar. Mit fortschreitender Behandlungsdauer werden Unterbehandlung und ein ungewolltes Verlassen der langfristig angelegten Therapieregime und erforderlichen Wiederbehandlungen beobachtet. Die Ursache oder der konkrete Auslöser ist im Einzelfall nicht zu identifizieren. Behandler können aber Maßnahmen ergreifen, um die Adhärenz der Patienten zu unterstützen und einer mangelhaften Persistenz entgegenzuwirken. Den konkreten Fragen und Auslösern hat sich eine Runde von Experten gewidmet.

Bisher ist es der zeitlich begrenzten Wirkdauer aller VEGF-Inhibitoren („vascular endothelial growth factor“ [VEGF]) geschuldet, dass eine häufige Nachkontrolle und Wiederbehandlung im Rahmen der IVOM-Therapien erforderlich sind. Insbesondere für die Therapie der neovaskulären altersabhängigen Makuladegeneration (nAMD) muss die chronische Aktivität berücksichtigt werden, die trotz der Wirksamkeit der Präparate eine hochfrequente Behandlung zur Stabilisierung der Visusgewinne über Jahre erforderlich macht. Während in randomisiert kontrollierten Studien (RCTs) eine hohe Rate der Nachverfolgung und Weiterbehandlung – insbesondere mit häufigen, oft sogar monatlichen IVOMs – erreicht werden konnte [6, 25], wurde im Versorgungsalltag ein frühes Ausscheiden vieler Patienten beobachtet [17]. Allein das steigende Alter kann relevante Hürden wie Begleiterkrankungen, Einschränkungen der Mobilität oder den Verlust der Motivation nach sich ziehen, die dem Verbleiben in der ophthalmologischen Betreuung entgegenstehen. Zudem beschränken sich RCTs und vergleichende Studien oft nur auf die ersten Jahre der Behandlung [34], die Abbruchraten dürften aber nach mehrjähriger Vorbehandlung und Therapiewechseln ansteigen [21]. Obwohl die Augenheilkunde strukturell in der Breite und Qualifikation exzellent aufgestellt ist, stellen die hohe Prävalenz der nAMD und die Notwendigkeit der hochfrequenten Behandlung Angehörige, Patienten, Kostenträger und Mediziner vor große Heraufforderungen. Inzwischen stehen weniger Finanzierungslücken einer besseren Umsetzung der mehrjährigen IVOM-Therapie entgegen als Flaschenhälse wie bürokratischer Aufwand, eingeschränkte Reservekapazitäten und organisatorische Stolpersteine (Transportfrage, Terminkollisionen) [28].

Bereits die erste Injektion nach der Diagnose findet häufig verzögert statt [60]. Schon 3 Monate nach Beginn der IVOM-Therapie wurde bereits ein Drittel der Patienten nicht so behandelt, wie dies von den Fachgesellschaften empfohlen wird [11]. Nach 6 Monaten waren es sogar zwei Drittel [13, 27]. Nur etwa 30 % der Patienten befinden sich nach den ersten 2 Jahren noch in Behandlung [2, 7]. Als Abweichungen vom ursprünglich geplanten Therapieregime werden Non-Adhärenz (NA) und Non-Persistenz (NP) (Tab. 1; [14, 35]) unterschieden. „Adhärenz“ sollte den veralteten obsoleten Begriff der „Compliance“ vollständig ersetzen, der wörtlich übersetzt („Gefügigkeit“) zu stark auf eine kritiklose Einhaltung der verordneten Therapie beschränkt blieb [1]. Gerade in die Planung einer anspruchsvollen Behandlung sollte der Patient mit seinen individuellen Problemen und Möglichkeiten einbezogen werden. Die Weltgesundheitsorganisation (WHO) unterscheidet 5 Dimensionen (Tab. 2; [56]).

**Table 1:** 

Bezeichnung	Definition
Adhärenz	Ein Patient kann als *vollständig adhärent* zur intravitrealen Therapie betrachtet werden, wenn er – jeden geplanten Kliniktermin^a^ (Behandlung oder Monitoring) wahrnimmt und sich jeder Behandlung oder Überwachung nach Empfehlung der behandelnden Ärzte unterzieht Ein Patient kann als *adhärent* zur Anti-VEGF-Therapie betrachtet werden, wenn er – nicht mehr als einen Behandlungs- oder Kontrollbesuch^a^ versäumt, der nach ärztlicher Empfehlung über einen Zeitraum von 1 Jahr geplant ist
Non-Adhärenz	Ein Patient kann als *non-adhärent* zur intravitrealen Therapie angesehen werden, wenn er – 2 oder mehr Behandlungs- oder Kontrollbesuche^a^ versäumt, die nach ärztlicher Empfehlung während des Zeitraums eines Jahres geplant sind
Persistenz	Ein Patient kann als *persistent* in Bezug auf die intravitreale Therapie angesehen werden, wenn er – die Behandlung oder das Monitoring gemäß der ärztlichen Empfehlung fortsetzt und den letzten Termin wahrgenommen hat Persistenz setzt keine Adhärenz voraus
Non-Persistenz^b^	Ein Patient kann als *non-persistent* in Bezug auf die intravitreale Therapie betrachtet werden, wenn – er aus irgendeinem Grund während eines Zeitraums von 6 Monaten keinen Behandlungs- oder Monitoringbesuch wahrgenommen hat – aus irgendeinem Grund für einen Zeitraum von 6 Monaten keine Folgetermine festgelegt werden

^a^Ein Besuch gilt als versäumt, wenn der empfohlene Termin aus irgendeinem Grund um mehr als 2 Wochen überschritten wird

^b^Der erste Tag des 6‑Monats-Intervalls nach dem zuletzt wahrgenommenen Termin sollte als Beginn der Non-Persistenz angegeben werden

**Table 2:** 

Kategorie	Unterkategorien
Gesundheitssystem	Administrative Probleme Zugang zur Behandlung, z. B. Verfügbarkeit von Terminen Distanz zum Behandlungsort Umzug oder Wechsel des Patienten zu einem anderen Arzt Andere
Sozialökonomie	Mangel an Transportkapazitäten, Transportprobleme Verfügbarkeit von Angehörigen und/oder Pflegepersonal (z. B. Begleitung) Direkte Kosten, Erstattungsfragen Indirekte Kosten (z. B. Parkgebühren, Produktivitätsverlust)
Krankheitsbezogen	Behandlungserfolg (nach Einschätzung des Patienten) Behandlungsfehler (nach Einschätzung des Patienten) Behandlungserfolg/stabile Krankheit (nach ärztlicher Beurteilung) Behandlungsfehlschlag, fehlender Nutzen, Eintreten der Abbruchkriterien (nach ärztlicher Beurteilung) Auftreten von Kontraindikationen Schlechter Ausgangsvisus Andere
Therapiebezogen	Beschwerden im Rahmen der Behandlung Auftreten eines unerwünschten Ereignisses Auftreten von Unverträglichkeiten Angst vor Injektionen, Spritzenphobie Andere
Patientenbezogen	Motivationsverlust des Patienten Okuläre Begleiterkrankungen Nichtokulare Komorbiditäten, allgemeine Gesundheit, Allgemeinzustand Andere

## Erfahrungen aus dem Alltag für den Alltag

Inzwischen gibt es eine wachsende Anzahl an Studien, die sich mit den Gründen beschäftigen [14]. Dabei steht eine patientenzentrierte Betrachtung im Vordergrund [5, 50, 55]. Obwohl Daten zur Zufriedenheit (Retinopathy Treatment Satisfaction Questionnaire [RetTSQ]) und Wünsche der Patienten bekannt sind [22, 39, 44], gibt es kaum interventionelle Studien, die die Wirksamkeit von Maßnahmen auf eine bessere Therapietreue belegen. Retrospektive Daten müssen aufgrund des Verzerrungspotenzials mit Vorsicht interpretiert werden [61].

Eine Gruppe von Experten (Vertreter von Universitätskliniken, niedergelassenen Praxen und Ophthalmochirurgen) hat Einflussfaktoren aus der eigenen Erfahrung und der Literatur zusammengetragen und bewertet. Die Empfehlungen beziehen sich auf die IVOM-spezifischen Einflussfaktoren. Die mögliche Optimierung der IVOM-Therapie ist Chance und Verpflichtung zugleich, das Potenzial für den Erhalt der Sehkraft durch eine konsequente Umsetzung voll auszuschöpfen [40].

## Erfassung und Monitoring von Qualitätsparametern

**Figure 1:**
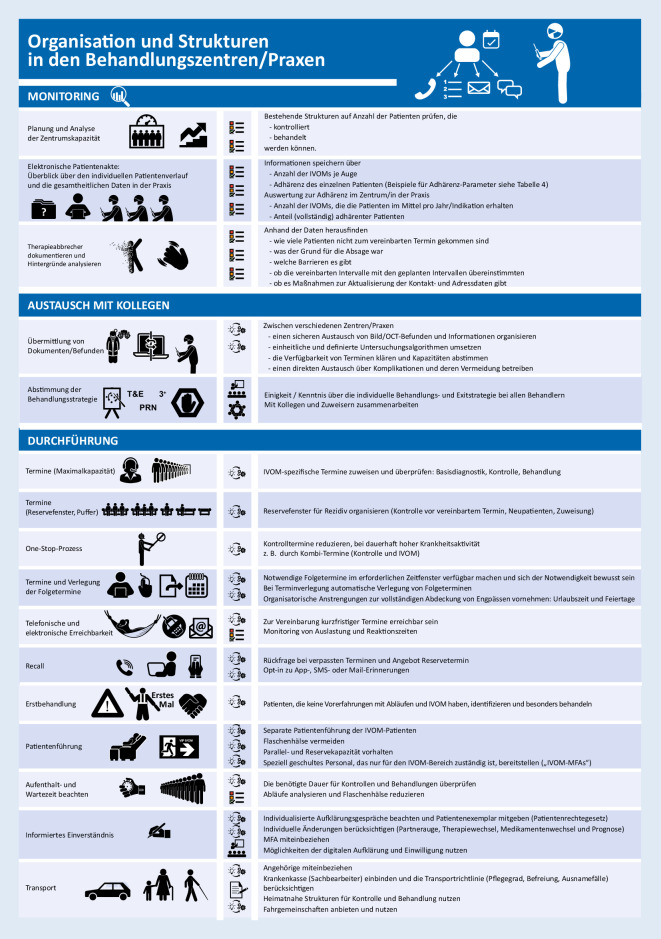

Im Kontext der IVOM-Therapie können die behandelnden Ärzte die Anzahl verabreichter Behandlungen – ähnlich anderen invasiven Therapien – kennen und analysieren, während bei Augentropfen und oralen Medikamenten eine große Dunkelziffer in Bezug auf die Therapietreue verbleibt [32]. Ophthalmochirurgen wissen, welche Wirkstoffdosis ein Patient erhalten hat, wann und wie oft eine IVOM-Behandlung unterbrochen, verzögert oder beendet wird. Weil nur erkannte Probleme bearbeitet werden können, ist es unabdingbar, sich selbst durch eine regelmäßige Auswertung das genaue Ausmaß und die Rate der Therapieabbrecher bewusst zu machen (Abb. 1; [59]).

Ein gutes Ansprechen scheint sich auf die Adhärenz stärker als der Ausgangsvisus auszuwirken. Selbst wenn die Therapie für eine Subgruppe wegen einer fehlenden Visusprognose (≥0,05) zu Recht beendet wird, verschlechtert die Unterbehandlung der nAMD für die große Mehrheit wegen des desaströsen Spontanverlaufs das Ergebnis [9]. Nahezu ausnahmslos zeigt sich daher die Anzahl verabreichter IVOMs als prädiktiver Faktor (Tab. 3). Unter vielen untersuchten Faktoren stellte sich bereits in der AURA-Studie die Anzahl der Behandlungen pro Patient/Jahr als entscheidende Größe heraus [27]. Selbst im Umfeld einer kontrollierten Studie (CATT) war das Verpassen oder die Verzögerung einer Visite bereits mit einem relevanten Sehverlust verbunden [43]. Daher kann ein Abgleich des geplanten mit dem tatsächlichen Behandlungsintervall Sinn machen [58]. Eine Unterbehandlung kann aber auch darin begründet sein, dass Aktivitätskriterien trotz erfolgter OCT-Untersuchung übersehen werden [8]. Hier ist also auch ein zweiter Blick auf die Bilder z. B. als Stichprobe oder im späteren Verlauf angeraten.

**Table 3:** 

Parameter	Einheit	Definition	Referenz
Anzahl IVOMs pro Auge	*n* /Zeitintervall	Anzahl verabreichter IVOM pro Behandlungsjahr	[27]
Ausfallereignis	*n* /Zeitintervall	Anzahl verpasster Visiten (pro Beobachtungszeitraum)	[43]
Kontrolldichte/Regelmäßigkeit	Zeitintervall	Durchschnittliches Zeitintervall zwischen allen Visiten (pro Beobachtungszeitraum)	[43]
Abreißen/Pausen	Zeitintervall	Längstes Intervall zwischen 2 Visiten (in einem Beobachtungszeitraum)	[43]
Mindestabdeckung	% oder Wahrheitswert	Unterbrechung: mindestens 1 Visite pro Quartal (in einem Beobachtungszeitraum)	[43]
Anzahl Patienten mit Behandlungslücke	*n*	Anzahl von Patienten mit ungewollter Pause (definiert ≥3 Monate vs. geplant 1 Monat)	[58]
Behandlungsverzögerung	*n*	Anzahl von Patienten mit ≥2 Visiten ohne Wiederbehandlung trotz Aktivität nach OCT-Kriterien	[8]

*n* = Anzahl

Die genannten Parameter lassen sich für jede Indikation ansonsten auf der Basis extrahierter Abrechnungsdaten (Besuchs‑, Behandlungsdaten) für jede einzelne Klinik und Praxis analysieren. Das Monitoring erlaubt die Anpassung der Kapazitäten und Reserven für die Zukunft [37]: Schließlich muss geklärt sein, wann neue Patienten oder Menschen mit einem Rezidiv zeitnah eine IVOM erhalten können [29]. Die ganzjährige Behandlungs- und Therapiemöglichkeit muss auch verdichtete Abläufe rund um Feiertage und Urlaubszeiten berücksichtigen [36].

## Professionelle Zusammenarbeit und Befundaustausch

Spätestens nach Aufnahme der optischen Kohärenztomographie (OCT) in den Einheitlichen Bewertungsmaßstab (EBM) ist eine heimatnahe (Mit‑)Betreuung einfacher geworden [17]. Portalbasierte Strukturen des Bildaustausches und deren Befundung können die Latenzzeit zwischen Therapieindikation und IVOM/Re-IVOM deutlich unter 14 Tagen halten [48].

Neben der Frage, wie die relevanten Schnitte eines OCT-Volumenscans in ausreichender Qualität zwischen Augenärzten ausgetauscht werden können, muss abgestimmt werden, welches Behandlungsregime (pro re nata, „treat & extend“ oder „observe & plan“) gewählt, welches Präparat verabreicht und wie die weiteren Termine organisiert werden sollen [11].

## Berücksichtigung relevanter Barrieren bei der Durchführung

Das Risiko, aus der Behandlung auszuscheiden, nimmt mit Alter sowie Anzahl der Vorbehandlungen und Kontrolluntersuchungen zu [59]. Es wurde ein Unterschied zwischen den Behandlungsindikationen beschrieben: Patienten mit einem diabetischen Makulaödem (DMÖ) brechen die IVOM-Therapie signifikant früher und häufiger ab als Patienten mit einer nAMD oder Makulaödem nach venösem Gefäßverschluss (RVO) [12, 19, 59]. Außerdem kann eine einzelne Nebenwirkung, Schmerzempfindung oder Komplikation die Gefahr des unerwünschten Endes signifikant erhöhen [59]. Daher muss nicht nur die Prävention einer Infektion beachtet werden, sondern auch weniger dramatische Störungen der Befindlichkeit wie Verletzungen und Reizungen der Augenoberfläche müssen vermieden werden [33]. Schmerzen können durch technische Details der Durchführung, aber auch durch die Abfrage und das Bewusstsein im Vorhinein ausgeschlossen werden [47].

## Organisationseffizienz

Je länger die Patienten (und ihre Angehörigen, Begleiter oder Fahrer) durch die einzelnen Behandlungstermine zeitlich belastet werden, umso größer ist das Risiko einer Behandlungsunterbrechung oder sogar des Therapieabbruchs [52]. Daher sind spezifisch angepasste Termine sinnvoll, die sich an den Anforderungen der konkreten Abläufe (OCT, Befundung, IVOM) orientieren [10]. Die Strukturen sollten an die Prozesse angepasst werden, Nadelöhre durch wenige Untersuchungsgeräte und unzureichende Wartekapazitäten vor den Eingriffsräumen müssen vermieden werden. Nahezu alle EDV-Systeme erlauben es heute, die Aufenthaltszeit zu erfassen. Aber bereits die Nachfrage bei den Patienten kann ein realistisches Bild vermitteln und die Zufriedenheit erhöhen [15]. Aus organisatorischer Sicht gibt es gute Argumente für die Reduktion der Patientenbesuche, z. B. indem eine beidseitige IVOM an einem Termin angeboten wird.

Für die Festlegung der Termine muss das Vorgehen sowohl für Patienten und ihre Bezugspersonen als auch für das Team nachvollziehbar bleiben. So sollte Klarheit über die Wiederbehandlungsstrategien bestehen [34]. Der theoretische Vorteil der Treat & Extend-Strategie, die Zahl der notwendigen Besuche durch eine strikte Kombination von Vorstellung und Wiederbehandlung zu verringern, zahlt sich streng genommen nur aus [3], wenn die IVOM auch wirklich im Rahmen der Vorstellung (1-Stop-Strategie) verabreicht wird. Dadurch kann für Patienten und ihre Angehörigen der Aufwand der Anfahrt zum Behandlungszentrum entfallen [24]. Ein entsprechendes Vorgehen hat insbesondere bei langen Anfahrtswegen und unilateraler Erkrankung Vorteile [2, 45, 57]. Außerdem können die Kapazitäten für notwendige Behandlungen besser geplant und vorgehalten werden.

## Vertrauen und Beziehungspflege

**Figure 2:**
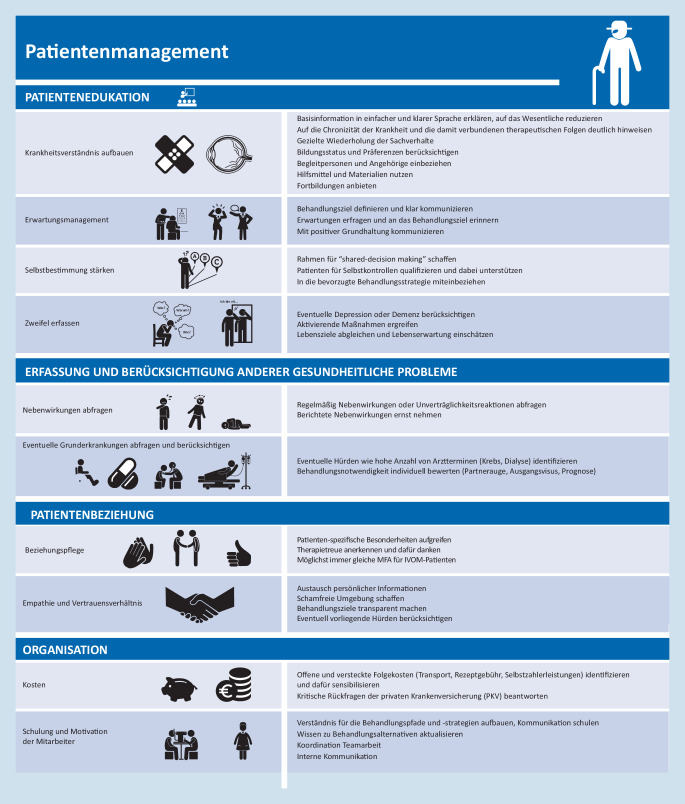

In der Kommunikation mit Patienten sollten unterschiedliche Formate verwendet und bewusst Redundanz kongruenter Informationen beachtet werden (Abb. 2; [18]). Die Einhaltung von Terminen zur Nachkontrolle bzw. Wiederbehandlung kann konsequent unterstützt werden. Mit steigender Anzahl der beteiligten Mitarbeiter und Ärzte wächst – ohne ausreichende Abstimmung – die Gefahr von Widersprüchen. Abgesehen von Terminkarten und Behandlungsplänen können heute auch digitale Formate verwendet werden [26]. So erlauben Apps neben der Vermittlung von Krankheitswissen den Eintrag von Terminen und Daten. In anderen Bereichen der Medizin wurden positive Erfahrungen mit Messaging-Systemen (SMS oder Mail) gemacht, die im Vorfeld an einen Termin erinnern [31]. Hier können der genaue Wortlaut und die richtige Ansprache der Textnachrichten von großer Bedeutung sein, um die größte Wirksamkeit (Inanspruchnahme, Absprache eines Ersatztermins) zu erreichen [4]. Während Erinnerungsanrufe in Studien und einem anderen Kontext weit verbreitet sind, ist die Verbreitung in der Augenheilkunde noch gering.

## Patientenwissen

Frühere Studien zeigen, dass das Wissen über die Erkrankungen der Netzhaut oft – selbst nach zahlreichen IVOM-Behandlungen – noch große Lücken aufweist [16]. Ein umfassendes Verständnis ist aber nicht nur für die gemeinsame Diskussion der Behandlungsziele wichtig (Patientenautonomie), sondern auch wichtige Voraussetzung für die intrinsische Motivation, den erwünschten Erhalt der Sehkraft in konkretes Handeln zu überführen. Daher sind Patienten und ihre Angehörigen in den gesamten Prozess der Behandlung einzubeziehen [41]. Gerade zu Beginn der Behandlung sind die Inhalte der allgemeinen Behandlungsaufklärung von Alternativen und Spontanverlauf bis hin zu Therapiezielen, Terminen und Warnsymptomen der Endophthalmitis sehr umfangreich. Es muss beim ersten Gespräch vor Therapiebeginn den Patienten und Angehörigen klargemacht werden, dass es sich um eine ggf. lebenslange Behandlung mit Kontrolluntersuchungen handelt. Eine internationale Stichprobe des Informationsangebots im Internet hat zwar qualitativ gute Angebote durch institutionelle Seiten identifiziert, aber auch verkürzte und fehlerhafte Angaben von privaten Einrichtungen gefunden [46]. Laien dürften allerdings nur bedingt die Qualität von Internetforen und beworbenen Angeboten der Alternativmedizin beurteilen können. Zudem bieten die Inhalte nicht den Bezug zum eigenen Krankheitsgeschehen und unterstützen die Motivation zur Fortsetzung der Therapie nur begrenzt. Eine Gesprächsatmosphäre mit aktiven Anteilen der Patienten schließt auch die Kontrolle des Patientenwissens und die Einbindung offener Fragen ein.

## Motivation und Beziehungspflege

Die erwünschte Gesundheitskompetenz ist nicht allein durch Faktenwissen zu Erkrankung und IVOM-Therapie definiert. Vielmehr müssen persönliche Sorgen und Ziele stärker beachtet werden. Engagement kann durch positive Aussagen und die klare Formulierung des Behandlungsziels verstärkt werden. Augenkontakt kann Vertrauen verstärken. Zahlreiche Arbeiten haben das Ausmaß der psychischen Ausnahmesituation und Belastung aufgezeigt, die Erstdiagnose und die erlebte Sehbehinderung für die Betroffenen und ihre Angehörigen bedeuten [23, 42, 54]. Depressive Reaktionen und eine ängstliche Gefühlslage sind entsprechend häufig anzutreffen [49]. Empathie und eine ausreichende Reflexion dieser emotionalen Belastung sind wichtige Voraussetzungen, um zu den Patienten durchzudringen und eine Basis für ein langjähriges Vertrauensverhältnis zu schaffen.

Patienten sind vor der ersten IVOM von Angst und Ungewissheit betroffen. Auf die besonderen Anforderungen kann spezifisch eingegangen werden: Indem gerade solche Patienten in der großen Gruppe einer IVOM-Sprechstunde identifiziert und „markiert“ werden, ist es möglich, ihnen eine Sonderbehandlung zuteilwerden zu lassen. Das Halten der Hand, ein beruhigendes Gespräch oder das vorsichtige Ankündigen der einzelnen Schritte kann Ängste vor der ersten Behandlung abbauen.

Betroffene und ihre Bezugspersonen sprechen nicht von sich aus an, wenn Unverständnis über eine Medikamentenzuzahlung besteht [53]. Die Sorgen, anderen Menschen zur Last zu fallen, oder die Unsicherheit, medizinische Maßnahmen könnten sich allein wegen des Alters nicht mehr lohnen, werden nicht aktiv artikuliert [5]. Oft können sensible Worte hier beruhigen. Tipps für ein unterstützendes Verhalten können Orientierung geben. Das anerkennende Verständnis um die Belastungen kann helfen. Stehen die Transportkosten im Vordergrund, können im Einzelfall Behandlungsstrategien angepasst, aber auch die Möglichkeiten der Richtlinie über die Verordnung von Krankenfahrten, Krankentransportleistungen und Rettungsfahrten (§ 92 Absatz 1 Satz 2 Nummer 12 SGB V, Transportrichtlinie) ausgeschöpft werden [20]. Obwohl die IVOM (noch) nicht im AOP-Katalog enthalten ist, kann im begründeten Einzelfall Rücksprache mit Kostenträgern gehalten werden. Die Fahrt zum Behandler darf keinesfalls der wesentliche Grund für einen Therapieabbruch sein. Abgesehen vom Einzelschicksal käme das der Allgemeinheit (Sozialversicherung) teurer zu stehen.

## Interaktion und Redundanz

Selbstverständlich muss der tatsächliche Patientenwunsch evaluiert werden. Kommunikative Techniken zur Unterstützung des Patienten und Verstärkung sinnvollen Verhaltens sollten genutzt werden [51], ohne dass Betroffene gegen ihren Willen bevormundet werden. Je besser man in und nach den Gesprächen die Motivation und Überlegungen der Betroffenen kennt [30], umso zielgerichteter können Hilfe und Unterstützung erfolgen. Durch Schwerhörigkeit, hohes Alter und sich wiederholende Gespräche ist die Kommunikation nicht frei von Frustration. Die Interaktion mit Patienten bleibt jedoch das Schlüsselelement, um die Adhärenz zu verbessern.

## Umsetzung und Veränderungsprozesse

Nur mit stetigen Veränderungsprozessen können effizientes Arbeiten und größere Zufriedenheit erreicht werden [38]. Jede einzelne Maßnahme kann helfen, Widerstände und Stolperfallen zu identifizieren. So wird sichergestellt, dass der Abbruch der Therapie kein unbemerktes Begleitphänomen bleibt. Strukturelle Veränderungen und die bewusste Beziehungspflege können den langfristigen Erfolg verbessern.

## Fazit für die Praxis

Es ist ärztliche Aufgabe, unerwünschte Therapieabbrüche im Rahmen der IVOM-Therapie zu vermeiden. Organisatorische Maßnahmen können das Auftreten von Sollbruchstellen verringern. Die patientenseitige Perspektive ist zu berücksichtigen. Mit dem Bewusstsein um die relevanten Faktoren können auch die nichtärztlichen Mitarbeiter (medizinische Fachangestellte, Op-Personal) die Therapietreue unterstützen. Zukünftige Studien müssen aber erst noch bestätigen, ob eine effiziente Gestaltung der Abläufe durch technische Hilfsmittel, digitalen Befundaustausch und Schulungen aller Mitarbeiter über Behandlungsregime und notwendige Folgetermine eine Verbesserung der Adhärenz erreichen kann.
